# Pervasive systemic drivers underpin COVID-19 vulnerabilities in migrants

**DOI:** 10.1186/s12939-021-01487-2

**Published:** 2021-06-22

**Authors:** Ferdinand C Mukumbang

**Affiliations:** Department of Global Health, School of Medicine, University of Washington, Seattle, USA

**Keywords:** COVID-19, COVID-19 nationalism, xenophobic stigma

## Abstract

Asylum seekers, refugees and undocumented foreign nationals have always been identified as a vulnerable population owing to the longstanding structural barriers and inequalities that they continually face. Their vulnerabilities have become more conspicuous and exacerbated since the advent of the Coronavirus disease of 2019 (COVID-19) pandemic. The plights of these migrants around the world, in the COVID-19 era, are therefore underpinned by not-so-new but enforced, re-emerging and adapting pre-existing systemic inequality drivers. Long-standing and pre-existing systemic drivers such as nationalism and anti-migrant or xenophobic stigma, in the context of the COVID-19 pandemic, have metamorphosed into COVID-19 nationalism and COVID-19–related xenophobic stigma respectively, fomenting discriminatory and segregation-laden policies and programmes. Transformative changes of asylum policies taking holistic and systematic perspectives while fostering the involvement of migrants in government planning and policy processes to redesign better policies are required to tackle the pervasive systemic drivers that underpin COVID-19 vulnerabilities in the identified migrant groups.

## Introduction

People who are on the move, who have left their countries and have crossed borders constitute a critical group regarding the Coronavirus disease of 2019 (COVID-19). This is because they (a) can act as vectors by carrying the SARS-COV-2 virus to transit and host populations [1]; (b) experience greater socio-economic and socio-cultural consequences and disruptions [2]; (c) disproportionately die from the disease – COVID-19 mortality rates of migrants exceed those of their corresponding native-born populations [3, 4]. Migrants’ vulnerabilities are severely enhanced by the social determinants of health such as education, employment, social security and housing [5]. These vulnerabilities have been exacerbated and made conspicuous in the context of the COVID-19 pandemic and intensified for those living in countries or regions where welfare services, social safety nets and other social protection mechanisms are weak or even absent [6].

Debates around social inequalities in health and medicine have received renewed attention globally in the wake of the COVID-19 pandemic [7]. Disproportionate biomedical risk factors and social determinants contribute to COVID-19 health disparities and can be traced, in part, to a foundation of structural inequality – bias embedded in the culture of an organisation, institution or government, which provides advantages for some members and marginalises or produces disadvantages for other members. While structural inequalities were of increasing concern in social science and public policy debates before the advent of the COVID-19 pandemic [8], such pervasive systemic and structural inequities are intensified in many different ways in the era of the COVID-19 pandemic. These structural inequalities are operationalised as oppressive governance policies, which are happening in response to the COVID-19 pandemic in many countries [9].

COVID-19–related social inequalities are also characterised by social marginalisation and exclusion with social exclusion manifesting predominantly through unequal access to resources, limited political, social and economic participation and voice, and the denial of opportunities [10]. Such social marginalisation and exclusion are the conditions that most asylum seekers, refugees and undocumented foreign nationals around the world are facing [11, 12]. In this commentary, the root causes of social inequality vis-à-vis asylum seekers, refugees and undocumented foreign nationals in the context of COVID-19 are discussed, contributing to ongoing discussions on how the COVID-19 pandemic is showing the migrant-inequality narrative in an uncomfortable new light. We argue that the plights of migrants around the world, in the COVID-19 era, are underpinned by not-so-new but enforced, rejuvinated and adapting pre-existing structural inequality drivers. Two distinct but interrelated drivers are highlighted: COVID-19 nationalism and COVID-19–related xenophobic stigma.

### COVID-19 nationalism-driven inequality

Nationalism is a political ideology that enhances patriotism to one’s national identity and their nation-state. Nationalism incites one’s personal and emotional investment, supreme loyalty to their nationality or country including race, culture, religious beliefs, location, historical background, food and way of life [13]. Ethno-nationalism and xenophobia played a significant part in the populist messages and rhetoric of many nations especially in Western Europe [14]. Therefore, nationalism before the COVID-19 pandemic emerged from the “resistance of globalisation and the fear of foreign nationals or migration that threatens a utopic vision or in many cases – hope – for what life should mean for those sharing a nationality”[15]. The climax of the migration crisis, which occurred in the summer of 2015, intensified such nationalism with a rise in mostly right-wing populism across many parts of Europe and drove nation-oriented policy ideas [16]. Such anti-immigrant conditions boosted the negative discourse around migration, often considered to be grounded in the assumption that migrants are a threat to host countries’ culture and social systems. Consequently, there was an increasing tendency to advocate for closing the borders and trying to stop migration and enacting exclusionary laws to discourage the integration of migrants within these communities.

The inception of COVID-19 has been accompanied by a global rise of nationalism, reigniting and reinforcing some of the anti-immigrant, xenophobia, and isolationist policies and positions [17]. For example, Hill et al. [18] found that recent changes to federal immigration policies and current COVID-19 federal relief efforts in the USA have created additional barriers to health care for immigrants and their families. While the COVID-19 pandemic has encouraged a sense of global citizenship that focuses on common humanity and common global good [19], some countries are engaging in nationalist strategies in response to the pandemic, enacting exclusionary laws and policies that promote segregation and foster inequality against vulnerable populations such as asylum seekers, refugees and undocumented foreign nationals [20]. For example, one of the very first measures announced by the South African government to deal with the COVID-19 pandemic was to build a 40 km fence on the border between South Africa and Zimbabwe [21], a move considered to be an agenda of political opportunism enhanced by the xenophobic atmosphere relating to scares job opportunities for South African nationals. Even in situations where a new migration policy framework for the Southern African Development Community (SADC) Region was drafted to promote regular, safe and orderly migration in the region in the COVID-19 era, South Africa, Botswana and Namibia remained reluctant towards executing this policy to open up their borders further [22].

Nationalism in the context of the COVID-19 pandemic has strengthened authoritarian regimes and prompted the rise of a new kind of populism fuelled by virus conspiracy theories with defensive patriotism – COVID-19 nationalism [23]. For instance, in 2020, 193 countries closed down partially, restricting access to people from specific countries or closing some – but not all – of their land and sea borders in response to the COVID-19 outbreak. Some of these countries introduced targeted bans, restricting entry to specific groups of people based on their recent travel or nationality. The first travel bans targeted China and other people of Asian descent, followed by other countries that experienced the earliest known outbreaks of the novel coronavirus [24]. The occurrence of new-COVID-19 variances, which are purported to be more transmissible than previous variants in different countries has contributed to renewed fears of having people from these countries entering other countries such as the United States to America [25]. Travel ban laws are the most common forms of discrimination against migrants from such countries. Although such bans and border closures are predominantly applied as public health measures to curb the spread of diseases such as COVID-19, experts have debated the usefulness of travel restrictions to control the spread of pathogens as there is insufficient evidence to draw firm conclusions about the effectiveness of travel-related quarantine on its own [26] and they sometimes raise ethical and human rights concerns [27]. While border control measures may be justified today as a matter of urgency to combat the COVID-19 pandemic it has also renewed the waning agendas of populists pushing for border-fortification and has reinvigorated the politics of regionalism [28]. Indeed, Ikotun et al. [29] suggest that tools such as border closures are being used by some policymakers to institutionalise exclusionary measures post-COVID-19.

One of the actions taken by other countries instigated by COVID-19 nationalism at the inception of the pandemic was the mass repatriation and refoulement of many migrants, especially migrant workers. Such repatriations and refoulement in some instances were accompanied by work permit denial or revocation [30]. With the call for travel bans around the world, in early March 2020, most European countries placed such forced returns on hold. However, the situation rapidly generated frustration among policymakers who wanted to resume deportation operations as soon as borders started reopening [31]. While the return to the country of origin has also been predominantly voluntary, the uncontrolled opening of borders and lack of legal status before the pandemic has placed migrant workers in challenging situations, leaving them stranded [30]. These migrants are being subjected to differing levels of mobility restrictions and segregated or quarantined in overcrowded and unhygienic conditions away from nationals. In the context of the COVID-19 pandemic, such actions are driven by and reinforced through the invocation and normalisation of different narratives and rhetoric, which serve to discursively and physically separate, segregate and exclude migrants [30]. Such actions highlight some ways in which fears of migration and COVID-19 have caused some governments to react in ways that fundamentally undermine the effectiveness and humanity of countries’ responses to both [12]. For example, placing asylum seekers, refugees and undocumented migrants in under-resourced and crowded camps, which provide inadequate and overcrowded living arrangements to reduce the spread of the COVID-19 infection will rather increase the chances of COVID-19 outbreaks while undermining their human rights such as freedom from arbitrary detention [27]. The absence of basic amenities, such as clean running water and soap, insufficient medical personnel presence, and poor access to adequate health information are major problems in these settings [2] and on their own, constitute human rights violation.

Some governments consider the presence of asylum seekers and refugees, and in some instances undocumented migrants especially migrant workers, as “beneficial” to the country. The debate is usually around the fact that migrant workers are “needed to fill labour and skills shortages” and to “do the jobs that local workers cannot or will not do” [32]. Sceptics, including some Trade Unions and Civil Society Organisations, have argued that in many cases these stands are taken when employers prefer recruiting cheap and exploitable migrant workers over improving wages and employment conditions [32]. During the COVID-19 pandemic, migrants were made to live in perilous conditions and subjected to unkind (working) conditions such as unscrupulous working hours, sometimes exacerbated, compared to the pre-COVID-19 era [33]. For instance, in Canada, when the COVID-19 pandemic was declared in March 2020, temporary migrant agricultural workers were put under extreme pressure to avoid any disruptions in the food supply chain, which increased work demands leading to multiple reports of abuse [34]. The Canadian example illustrates how the COVID-19 pandemic has increased the risk of labour rights violations and vulnerability to exploitation for migrant, especially migrant workers [34].

While the arguments for implementing non-migrant friendly laws and practices relate to curbing the spread of the COVID-19 pandemic, the policies governing them are sometimes underpinned by nationalism characterised by the lack of engagement and consideration of this population in the countries’ economic, poverty, and hunger alleviation schemes [5]. Freier and Espinoza [35] observed that the governments of Chile, Peru and other countries in South America have reframed their migration governance in response to the COVID-19 pandemic by reinforcing a pre-COVID-19 securitised approach to migration that may translate into the increased socio-economic and legal exclusion of migrant and refugee populations. Zambrano-Barragán et al. [36] also found that migrants from Venezuela in both Colombia and Peru face legal and financial obstacles relating to discrimination, which impacts their access to healthcare concerning the COVID-19 pandemic. The systemic exclusion of migrants has implications for the complete eradication of the COVID-19 pandemic even in the light of available COVID-19 vaccines. Notably, systemic exclusion including the perceived low representation of their communities in vaccine trials contributes to enhancing the pre-existing vaccine hesitancy among migrant populations, which has been amplified in the context of the COVID-19 pandemic [37].

### Xenophobic stigma and COVID-19

Xenophobic stigma relates to negative feelings and attitudes and behaviours toward migrants that have become popularized by unfounded claims that immigrants fuel crime, bring diseases, and enhance economic instability. Anti-immigrant or xenophobic policies and rhetoric are directly related to the societal stigmatisation of migrants [38]. There is a long-established pattern of linking minorities, racial groups, and specific communities to disease. In particular, immigrants have been stigmatized as the origin of a wide variety of physical and societal ills including diseases [39]. Exclusionary nationalism also often equate specific groups with diseases themselves [17]. According to Su and Shen [20], when governments’ nationalist policies are aligned with the ideology of citizens who support those nationalist governments, these citizens tend to support the policies irrespective of the discriminatory nature of such policies. The citizens' endorsement of such exclusionary policies may also manifest in discriminatory behaviours such as social stigma.

In the context of a pandemic, a group of people is labelled, stereotyped, discriminated against, treated separately, and/or experience loss of status because of a perceived link with the disease [40]. During the onset of the COVID-19 pandemic, with growing news that the first case was reported in Wuhan, China; racist vitriol and xenophobic incidents became pervasive globally especially towards people of Asian descent [41]. These xenophobic reactions included social avoidance and being confronted concerning COVID-19 [42]. While xenophobia is endemic in many countries and societies, it can be exacerbated during times of public emergencies such as is observed in the COVID-19 pandemic. Recently, the COVID-19–related stigma is being directed towards migrants from countries where new variants of the SARS-COV-2 virus are being identified such as South Africa, the United Kingdom, Brazil and most recently, India. Such stigma is enhanced by naming the variants after the countries or places where they were first identified [43]. In the context of COVID-19 as with other pandemics, stigma is associated with a lack of knowledge about how COVID-19 spreads, a need to blame someone, fears about disease and death, and gossip that spreads rumours, myths, and conspiracy theories [40].

In disease-related stigmas, sometimes people who do not have the disease in question but share other characteristics with the original targeted group may also become stigmatised. In the context of the COVID-19 pandemic, social stigma started with Asians facing racism everywhere around the world, then extended against people of certain minority ethnic backgrounds as well as anyone perceived to have been in contact with the virus. A recent study conducted in India revealed that stigma is associated with being infected with COVID-19, or being in close contact with someone infected, along with belonging to a particular race, religion, and social class [44]. Evidence of social stigma has also been reported among migrants who have tested positive or living with people who have tested positive for COVID-19. In this way, migrants are being scapegoated for the spread of the COVID-19 pandemic especially if their country of origin is purported to have a relatively high incidence of the pandemic. Consequently, xenophobic tendencies are being fuelled by such pandemic-related stigma and in return, the pandemic-related stigma drives xenophobic tendencies. These xenophobic tendencies are manifested as spates of violence and protests, leading to disproportional victimisation of marginalised groups especially migrants [45].

It has been observed that increased uptake of immigrants is accompanied by an increase in certain infections and diseases. For instance, after receiving increasing numbers of refugees from Syria, there were increasing numbers of gastroenteritis cases, cutaneous leishmaniasis and malaria in Turkey [46]. Such observations allow populations to associate increase disease incidence and prevalence with immigrants or “the foreigner” [39]. With an increasing number of COVID-19 cases on the borders of different countries, many countries have seen a significant increase in the stigmatisation of anyone even remotely related to COVID-19. Misinformation, disinformation and rumours in the era of COVID-19 contribute to confusion, disorientation and risky or improper behaviour and some of this behaviour is manifested as stigma towards migrants [42]. In addition to the fear of the “unknown” and of the “other”, conspiracy theories around the COVID-19 pandemic exacerbate existing stigmatisation and consequently systemic xenophobia.

## Discussion

**Fig. 1:**
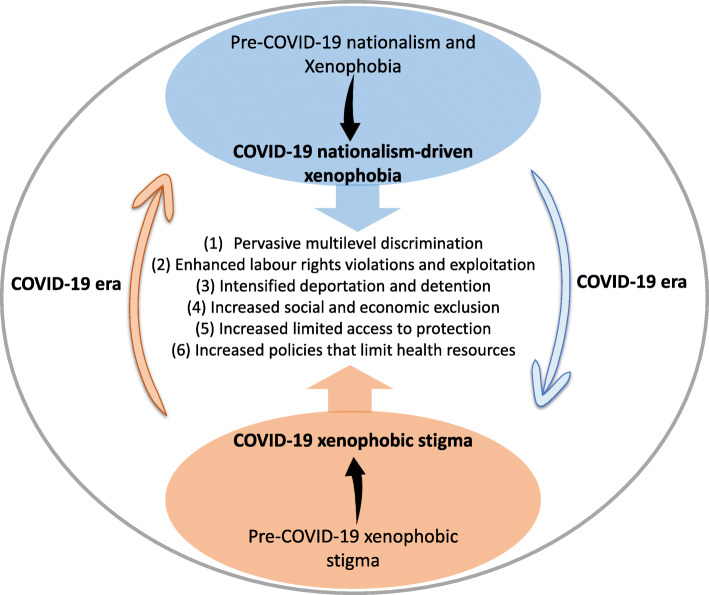
The relationship between COVID-19-related nationalism and xenophobic stigma.

COVID-19-related social stigmatisation makes it difficult for foreign migrants and asylum-seekers to access social protection and may exacerbate inequality, discrimination and exploitation [47]. The consequence of social stigma towards a social group is that these people will develop strategies, where possible, to hide the illness to avoid discrimination, which prevents them from seeking health care immediately and discourage them from adopting healthy behaviours. In this way, the COVID-19 pandemic and the fear of “the foreigner” shifts the migration and xenophobic rhetoric further, by expanding the focus to include the risk to individual health security. Indeed, when COVID-19–related nationalism and anti-immigrant or xenophobic stigma increase, three interrelated social and political processes manifest to harm the health of migrants: multilevel discrimination and stress, deportation and detention, and policies that limit health resources [38]. The relationship between xenophobic stigma and COVID-19-related nationalism and how they continue to enhance the vulnerabilities of migrants is illustrated in Fig. 1.

## Conclusions

Nationalist-driven decision making in the era of the COVID-19 pandemic has reignited and/or amplified sensitive social issues, including stigmatisation, discrimination, racism, injustice, and inequalities, exacerbating existing social and health disparities, especially regarding migrant health. Long-standing and pre-existing structural drivers such as nationalism and xenophobic stigma, in the context of the COVID-19 pandemic, have transformed into COVID-19 nationalism and COVID-19–related xenophobic stigma. These COVID-19 induced structural barriers have advertently or otherwise engendered discriminatory and segregation-implied policies and programmes. This new migration rhetoric also has longer-term implications for socioeconomic inclusion and social cohesion in societies receiving immigrants.

Transformative changes of asylum policies taking holistic and systematic perspective while fostering the involvement of migrants in government planning and policy processes to build back better policies are required to tackle the pervasive systemic drivers that underpin COVID-19 vulnerabilities [48]. Addressing the vulnerabilities experienced by migrants during the COVID-19 era and beyond also requires concerted and coordinated efforts from various stakeholders and requires removing the structural inequality barriers such as nationalism ideologies enforced by political opportunism, existing xenophobic tendencies and targeted discriminatory practices. Global stakeholders must also continue to support migrants and ensure that Civil Society Organisations active in the area of migration can continue to do their work by providing them with funding, and other resources, and ensuring that an appropriate framework is in place in which they can operate.

## Data Availability

Not applicable.

## Abbreviations

COVID-19: Coronavirus disease of 2019.
SADC: Southern African Development Community.
